# Potential Utilisation of Dark-Fermented Palm Oil Mill Effluent in Continuous Production of Biomethane by Self-Granulated Mixed Culture

**DOI:** 10.1038/s41598-020-65702-w

**Published:** 2020-06-08

**Authors:** Safa Senan Mahmod, Azratul Madihah Azahar, Abdullah Amru Indera Luthfi, Peer Mohamed Abdul, Mohd Shahbudin Mastar, Nurina Anuar, Mohd Sobri Takriff, Jamaliah M. D. Jahim

**Affiliations:** 1 Research Centre for Sustainable Process Technology (CESPRO), Faculty of Engineering and Built Environment, Universiti Kebangsaan Malaysia, 43600 UKM Bangi, Selangor Malaysia; 2 Chemical Engineering Programme, Faculty of Engineering and Built Environment, Universiti Kebangsaan Malaysia, 43600 Bangi, Selangor Malaysia

**Keywords:** Environmental sciences, Chemistry, Energy science and technology, Engineering

## Abstract

Two-stage anaerobic digestion of palm oil mill effluent (POME) is a promising method for converting the waste from the largest agricultural industry in Southeast Asia into a clean and sustainable energy. This study investigates the degradation of acid-rich effluent from the dark fermentation stage for the production of biomethane (BioCH_4_) in a 30-L continuous stirred-tank reactor (CSTR). The continuous methanogenic process was operated with varied HRTs (10 - 1 day) and OLRs (4.6–40.6 g_COD_/L.d^−1^) under thermophilic conditions. *Methanothermobacter* sp. was the dominant thermophilic archaea that was responsible for the production rate of 4.3 L_CH4/_L_POME_.d^−1^ and methane yield of 256.77 L_CH4_kg_COD_ at HRT of 2 d, which is the lowest HRT reported in the literature. The process was able to digest 85% and 64% of the initial POME’s COD and TSS, respectively. The formation of methane producing granules (MPG) played a pivotal role in sustaining the efficient and productive anaerobic system. We report herein that the anaerobic digestion was not only beneficial in reducing the contaminants in the liquid effluent, but generating BioCH_4_ gas with a positive net energy gain of 7.6 kJ/g_COD_.

## Introduction

In Malaysia, the palm oil industry has been recognised as the largest agricultural industry contributing to the economic development of the country. In 2018, for instance, the palm oil industry contributed to 8.7% of the country’s gross domestic product (GDP)^1^. Nevertheless, this industry is affected by environmental concerns since it is associated with the release of huge amounts of organic and inorganic contaminants. Hence, it is a priority to treat the by-products accordingly before releasing them into the surrounding lands and watercourses. Locally available oil palm biomass is rich in nutrients and can be utilised as an alternative energy source. During the palm oil milling operation, which mainly produces crude palm oil (CPO) and crude palm kernel oil (CPKO), a huge number of underutilised by-products are also generated. For each tonne of CPO, approximately 2.5 tonnes of palm oil mill effluent (POME), 0.9 tonnes of empty fruit bunches (EFB), 0.6 tonnes of mesocarp fibres, and 0.27 tonnes of shells are accumulated^2^. Currently, POME is treated with inefficient ponding system, while mesocarp fibres, shells, and EFB are used as fuels or as mulches^3^.

Several disadvantages are associated with conventional POME treatment such as the long hydraulic retention times (HRT), low treatment effectiveness, higher sludge accumulation, larger footprint, and release of copious amounts of greenhouse gases (GHG) in the form of CO_2_ and CH_4_ into the atmosphere, which is a big loss for energy recovery^4^. Conversely, the application of a well-organised, steady and inexpensive high-rate anaerobic treatment system has increasingly come under consideration^5^. POME has been identified as a source for many valuable bioproducts including biomass energy, namely biohydrogen (BioH_2_)^6–8^ and biomethane (BioCH_4_)^9,10^. Noting that by the year 2030, the world is predicted to consume 60% more energy than today^11^. Given the socio-political climate instability, urgent actions must necessarily be taken by environmental scientists around the world to find the best possible alternative energy sources.

The generation of biogas from organic materials is a significant part of the biogeochemical carbon cycle. Grossman and co-authors have explained that carbohydrates stored as cellular materials are metabolised to produce ATP. Meanwhile, the generated reduced pyridine nucleotide has to be oxidised again in order to activate the fermentation mechanisms, where the sugar content is transferred to acids and releases hydrogen gas, as an intermediate product, in what is known as dark fermentation (DF). Next, during anaerobic digestion (AD), methane is produced from the final stage of methanogenesis; these two stages, DF and AD, are referred to as the most vulnerable of all the phases^12^ and rely on the following parameters: temperature, pH, retention time, total ammonia nitrogen, and nutrient content of the reaction medium^13,14^. For further detail, the optimal pH for acidogenic growth lies in the range of 5 to 6 with an optimal HRT of 1 to 3 d. During acidogenesis, carbohydrates are first converted to hydrogen through the acetate and butyrate pathways. Hence, the high content of VFAs in the dark-fermented effluent renders its readiness for the second stage, in which the remaining organic content is anaerobically digested to methane via methanogens in the optimal pH range of 7 to 8 and optimal HRT of 15 to 20 d^15^. Based on the current state-of-the-art, energy analysts suggest that the two-stage fermentation process yielded a greater net energy recovery than the single BioH_2_-DF and single BioCH_4_-AD processes^16^. Recently, many studies have demonstrated the two-stage anaerobic fermentation for better utilisation of the locally-harvested substrate, herein POME, leading to less undigested waste, and more energy generation for better biogas economics^16–18^. These studies emphasize that, under the current state-of-the-art, the two-stage anaerobic digestion is a suitable method to reach high biogas yield and utilise the organic content in the substrate as much as possible.

The viability of several bioreactor designs has been investigated for the fermentative production of biogas using various substrates; such as anaerobic sequencing batch reactors (ASBR)^9,19^, continuous stirred-tank reactors (CSTR)^20–22^, anaerobic baffled reactors (ABR)^23^, anaerobic plug flow reactors (APFR)^24,25^, and up-flow anaerobic sludge blankets (UASB)^18,26,27^. Among these bioreactors, CSTR is commonly used for its simple design and enhanced mixing rate that offers good substrate-sludge contact with slight mass transfer resistance^25^. Many studies reported successful POME degradation using the CSTR with high BioCH_4_ generation; a modified CSTR with a deflector achieved a methane production rate (MPR) of 4.14 L_CH4_/L.d^−1^ and a yield of 0.27 L/g_COD removed_ with 82% COD removal at HRT of 3.3 d^21^, the maximum volumetric MPR and methane yield (MY) were 10.58 L_CH4_/d and 0.11 ${m}_{C{H}_{4}}^{3}$/kg_COD_, respectively at HRT of 5 d in an 11-L CSTR^22^. A similar study conducted by Nasir *et al*. that achieved COD removal of 71% at HRT of 3 d, and methane production rate of 3.96 L_CH4_/L.d^−1^ with corresponding methane yield of 260.3 L_CH4_//kg_COD removed_^9^.

The production of BioH_2_ which preceded BioCH_4_ production stage does not only harness additional energy, but also help in treating POME without reducing the amount of biomass and can effectively improve the start-up of BioCH_4_ production in the second stage. Based on our previous study of the first-stage of the DF process^28^, the hydrogenic effluent contains a good amount of VFAs; mainly acetic and butyric acids. Although two-stage anaerobic digestion is a well-established process, further improvement of the process is required. The objectives of this article were (1) to study the possibility of increasing OLR (shortening HRT) in the CSTR system; to improve BioCH_4_ productivity and contaminant removal from the hydrogenic effluent, such as COD, TSS, VSS and VFAs, (2) to explore the potential of methane producing granules (MPGs) on the stable production of BioCH_4_, and (3) to analyse the archaeal community under the optimal conditions for methane generation using PCR-DGGE method.

## Results

### Inoculum seed preparation

**Figure 1:**
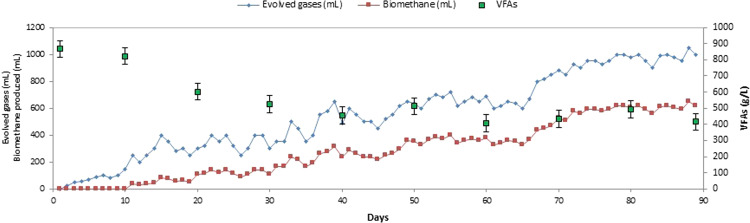
BioCH_4_ seed preparation in 1-L bioreactor for 90 days.

The BioCH_4_-producing seed sludge was initially acclimatised using the hydrogenic POME effluent in a 1-L sequencing batch bioreactor; the evolved gases were measured by water displacement method. The acclimatisation of the seed sludge was carried out for period of 3 months (shown in Fig. 1), notably, in the first 10 days only a small amount of CO_2_ was produced, with no BioCH_4_ nor BioH_2_ production. After day 10, VFAs started to reduce indicating that the microbial content was activated and had started to consume the acids; this was confirmed by the GC chromatogram for evolved gases that showed a detectable amount of BioCH_4_, until it reached the highest CH_4_ content of 62%. When the produced gases reached 1 ± 0.1 L/d, the effluent was collected and stored at 4 °C prior to being used as seed for the 30-L CSTR.

### BioCH_4_ production in CSTR

#### Kinetic study in batch mode CSTR

To understand BioCH_4_ production and substrate degradation, herein COD, during anaerobic digestion, kinetic study of sludge activity was conducted in a batch mode in 30-L CSTR. The kinetic parameters of modified Gompertz and first-order models are presented in Table 1, with initial total COD of 63.27 g/L. The first-order model gave a closer theoretical methane potential with an error of 5.36% to the experimentally produced methane (31.7 L), while the *M*_0_ given by the Gompertz equation recorded 22.7% difference relative to the experimental value. However, the Gompertz model showed an *R*^2^ of 0.01 higher than the first-order model, hence, Gompertz model fitted the BioCH_4_ system more than the first-order kinetic model.

**Table 1:** Kinetic parameters of 30-L batch operated CSTR BioCH_4_.

**Modified Gompertz**	
*M*_0_ (L)	24.50
*R*_*m*_ (L/d)	0.40
λ(d)	1.50
*R*^2^	0.98
**Modified First order equation**	
*k* (h^−1^)	0.02
*M*_0_ (L)	30.00
*R*^2^	0.97

In this study, the batch-wise operation was considered as a start-up for the continuous-mode fermentation, whereby 20% of the working volume of the CSTR was filled with pre-acclimatised seed sludge (prepared in the previous section), while the hydrogenic POME effluent occupied the remaining 80% of the working volume with the pH value adjusted at 7.

#### Effect of different HRTs on BioCH_4_ production

**Figure 2:**
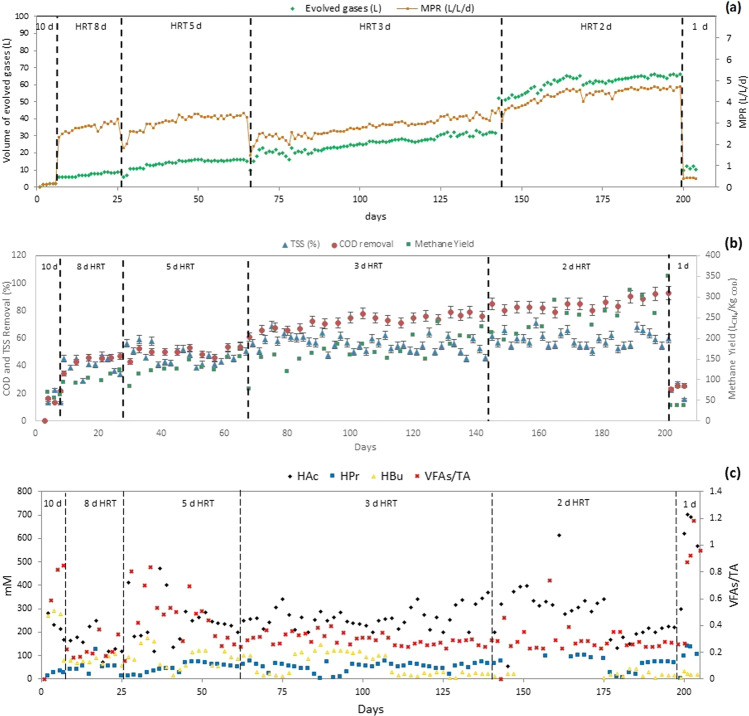
(**a**) Evolved gases and MPR profile, (**b**) TSS and COD removal (%) and BioCH_4_ Yield (LCH_4_/Kg_COD_), and (**c**) VFAs remained in BioCH_4_ effluent and VFAs:TA ratio correlation at different HRTs in 30-L CSTR.

After a stable biogas production was achieved in the start-up period, the feeding mode of CSTR was shifted to continuous, with varied HRT cycles. For the first 10 days, the CSTR was operated in batch mode as a start-up period. At HRT of 10 d, a layer of dirt, or scum, was observed in the headspace resulting in low biogas production and substrate digestion (Fig. 2). This was probably due to the high solid content in the feed with a relatively high portion of oil and grease, and a bad mixing mechanism in the reactor. Hence, a scum breaker (an extra blade paddle) was added to the top part of the stirrer, so that the evolved gases would not be trapped and prone to reaction failure.

At HRT of 8 d, a significant improvement in the biogas production was recorded, up to 55% CH_4_ in the evolved gas, with gradual reduction and disappearance of the scum layer. Further increases in the volume of the evolved gases and BioCH_4_ content (71–76%) were recorded with the decrease in HRT to 5 d that is equivalent to an OLR of 8.12 g_COD_/L.d^−1^. At HRT of 3 d cycle, a slight fluctuation in the BioCH_4_ production profile was recorded at the beginning of the cycle due to the increase in the substrate feeding rate to 8.33 L/d (OLR = 13.5 g_COD_/L.d^−1^), which affected the pH of the medium due to the accumulation of VFA that required some time for the methanogens to adapt to the new environment. The detected BioCH_4_ at HRT of 5 and 3 d were somehow similar, but the total volume of the evolved gases was higher at HRT of 3 d. The bioreactor operated at 2 d HRT for 2 months, with average production of BioCH_4_ 50–55 L/d with 80–82% of CH_4_ content, the production was considered stable. A drastic drop was recorded when the substrate feeding rate was doubled, at HRT of 1 d, where the bioreactor was fed with 12.5 L twice a day (=25 L/d).

As could be seen in Fig. 2(a,b), MPR and MY increased with the reduction of HRT. The average MY achieved were 61.97, 101.65, 127.00, 166.66, 256.77 and 18.51 (L_CH4_/kg_COD_) at HRT of 10, 8, 5, 3, 2 and 1 d, respectively. Both MPR and MY achieved their maximum values at HRT of 2 d, which is equivalent to OLR of 20.30 g_COD_/L.d^−1^. On the other hand, the operation at HRT of 1 d (equivalent to OLR of 40.60 g_COD_/L.d^−1^) caused a sudden drop in the BioCH_4_ production.

#### Effluent quality of BioCH_4_ CSTR

The chemical oxygen demand (COD) removal profile of the CSTR was stable within each cycle, as shown in Fig. 2(b). The average COD removal was 17.14, 43.52, 49.98, 72.41, 85.10, and 24.84% at HRT of 10, 8, 5, 3, 2 and 1 d, respectively. The lowest COD removal was recorded at HRT cycles of 10 d; indicating that the system was not efficient enough to degrade COD, which was a similar trend followed in the calculated MY and MPR. After the adjustment in the bioreactor design and removal of scum/dirt layer, the CSTR performance was improved together with the COD removal at lower HRTs. The stability of the system helped in maintaining high and stable COD removal at various HRTs. The highest COD removal and MY occurred at HRT of 2 d, which is equivalent to OLR of 20.3 g_COD_/L.d^−1^. However, at HRT of 1 d, the system was not efficient enough to degrade the overloaded COD, due to high OLR that was equivalent to 40.60 g_COD_/L.d^−1^ and the shift in the microbial community that led to washout.

The total suspended solids (TSS) removal followed the same trend as COD removal, as shown in Fig. 2(b). During the scum formation stage at HRT 10 d and microbial washout at HRT of 1 d, the TSS removal was less than 25% that was associated with the incomplete digestion of substrate and low biogas production. The average TSS removal was 16.67, 40.48, 47.92, 57.26, 64.25 and 21.97% at HRT of 10, 8, 5, 3, 2 and 1 d, respectively. The removal of substrate’s TSS and COD in the anaerobic digestion process represent the substrate degradation and contaminant deduction that is directly linked to the productivity of BioCH_4_.

Volatile fatty acids (VFAs), such as acetic acid and butyric acids, were the main by-products of BioH_2_- DF and the main substrate of BioCH_4_-AD. In correlation with the above discussion, the consumption of VFAs was directly linked to the BioCH_4_ generation. In Fig. 2(c), during the failed reaction at HRT of 10 d, the consumption of VFAs was very low, indicating that the reaction took a different pathway and started producing more acids instead of BioCH_4_. After the recovery in HRT of 8 d, a significant decrease in VFAs was recorded accompanied by the production of BioCH_4_. Results implied that increasing OLR improved the utilisation of VFAs by methanogens. Notably, accumulation in VFA occurred at OLR of 40.60 g_COD_/L.d^−1^ (HRT of 1 day) which led the pH of the medium to drop to less than neutral.

#### Characteristics of methane producing granules (MPGs)

**Figure 3:**
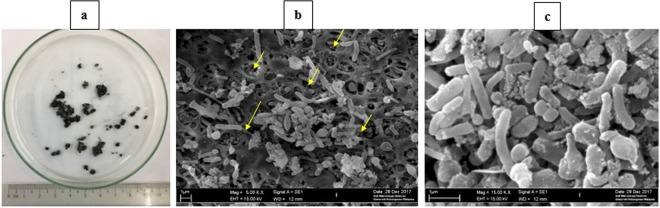
(**a**) MPGs formed in CSTR at HRT of 2 d, and MPG under scanned electron microscopy at magnification of (**b**) 5,000X, and (**c**) 15,000X.

After 100 days of the AD process and at HRTs of 3 and 2 d, small granules were found at the bottom of the CSTR. Unlike the HPGs that were formed in the first-stage of BioH_2_ production^28^, these MPGs were relatively small in size, fragile, and more regular/spherical in shape, as shown in Fig. 3(a).

The micrograph of the surface of the MPGs under various magnifications is presented in Fig. 3(b,c), where microbes of different shapes are attached to the hollow-surface of the MPG. The indication that there was more than one type of archaea responsible for BioCH_4_ production was proven by PCR-DGGE analysis. Microbes could easily grow inside these hollows providing better attachment between the archaea cells and the MPG surface. The SEM images showed some extracellular polymer substance (EPS), presented in yellow arrows in Fig. 3(b), imposing the quantification of EPS and protein content of MPGs.

#### Microbial community analysis

**Figure 4:**
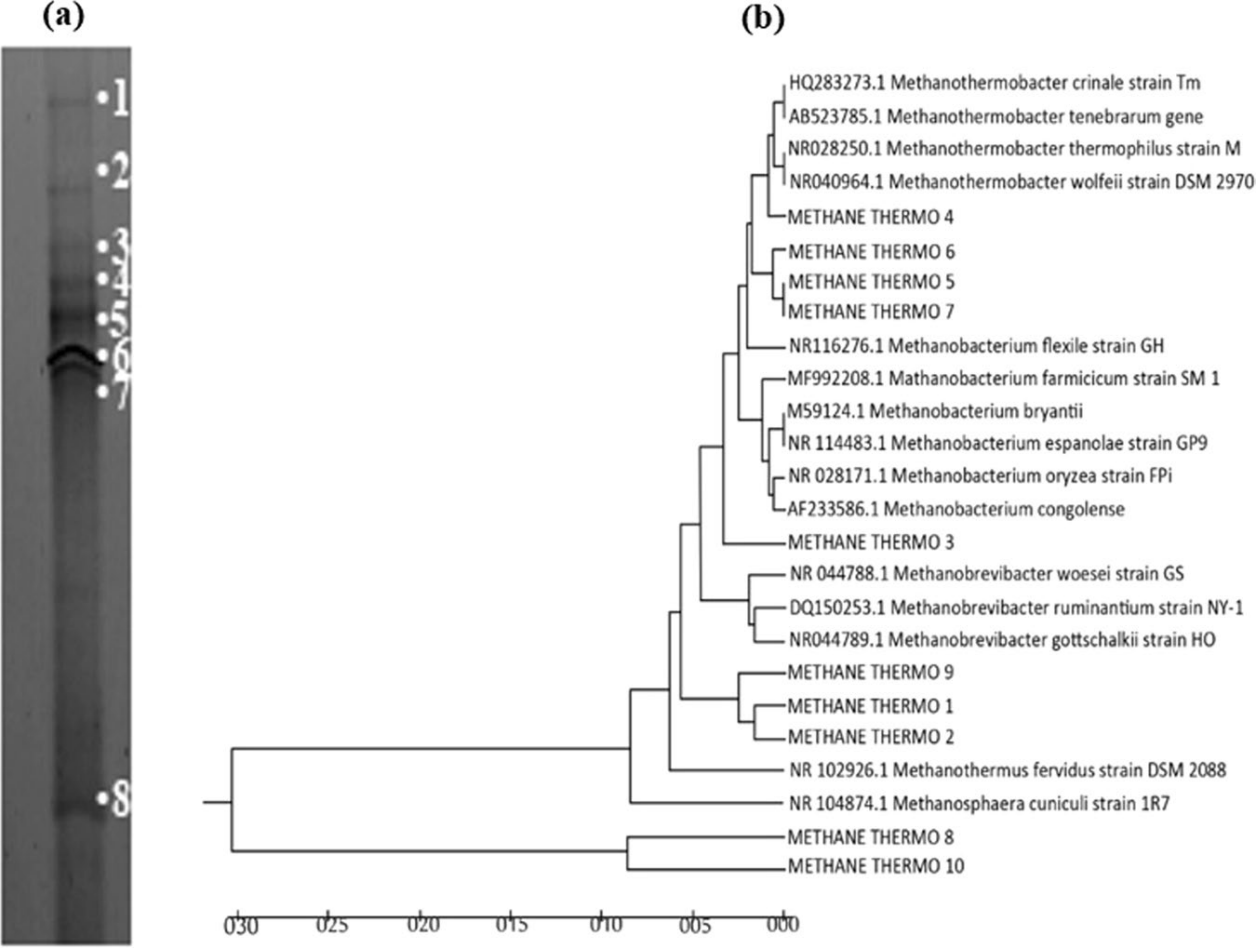
Microbial analysis of biomethane sludge sample at HRT of 2 d (**a**) DGGE profile of archaea, and (**b**) phylogenetic tree of the dominated thermophilic methane producing archaea, dominated by *Methanothermobacter* sp. (Band 4,5,6,7), then *Methanobacterium* sp. (Band 3), *Methanobrevibacter* sp. (Band 1,2), and uncultured *Methanobacterium* sp. (Band 8).

The archaeal community of thermophilic BioCH_4_ sludge sample at optimum HRT of 2 d was analysed for the archaeal 16S RNA gene using PCR-DGGE analysis. The bands in Fig. 4 represent *Methanothermobacter* sp., which is the dominant archaea, in Band 4, 5, 6, and 7. Also, Band 3, Band 1, 2, and Band 8 represent *Methanobacterium* sp., *Methanobrevibacter* sp., and uncultured *Methanobacterium* sp., respectively. Notably, all the detected microbes were exclusively methane producing archaea, and no *Clostridium* was found in the medium. Meaning that the reaction has successfully shifted from hydrogen-producing clostridium to methane-producing medium. The operation conditions of the CSTR and the acetate-rich substrate played an important role in the selective and reproducible effect on the archaeal community richness, evenness, structure and function.

## Discussion

In this study, the effluent from the first-stage (hydrogen-producing stage) anaerobic system was further utilised in the second-stage (methane-producing stage). The process started by preparing the seed inoculum from the 30-L CSTR, and studying the reaction kinetics of the batch system which helped in understanding the anaerobic digestion. In the continuous mode, the OLR and HRT were varied to determine the best operational conditions to achieve the optimum BioCH_4_ production while maintaining efficient substrate degradation. The substrate characteristics, reaction condition, and CSTR performance resulted in MPG formation that contributed in the system’s stability to be able to reach low HRT.

The kinetic parameters are important in predicting the time required for acclimatisation of the microbial community to the new environment, the duration of the AD process, and the biodegradability of the feed materials^29^. The lag phase (λ), which is an imperative process-kinetics parameter, explains or determines the efficiency of the AD process^30^. In this study, the estimated λ of 1.5 d could be ascribed to the nature of the substrate. Normally in BioCH_4_ systems, the lag phase is higher than the one obtained in this study, probably due to the efficiency of the acclimatisation process that resulted in productive microbial community and the less complex components in the POME hydrogenic effluent. For instance, raw POME was tested in a CSTR for biomethane generation with λ of 6 d, which was improved to λ of 1 d when co-digested with sludge enriched with methanogens^31^. Mamimin and co-authors examined the kinetic of co-digestion of POME with various types of oil palm biomass in two-stage anaerobic system, the lag phase for biomethane profile in POME sample was 0.34 d, which was increased by 2.6, 11 and 15 folds when combined with decanter cake, oil palm frond and empty fruit bunch, respectively. The solid residues in these three samples needed longer λ for adaption and initiating microbial growth^32^. Also, the high concentrations of protein and carbohydrate in the other lignocellulosic substrates require longer time to be anaerobically digested; such as lag phase of 4.20 d from swine manure and corn straw^25^, 5.13 d from potato starch^30^, and 8.07 d from animal dung^33^. Furthermore, the relatively long λ obtained in the abovementioned reports could be a result of the late response of the methanogens to the unexpected environmental conditions^34^. In this study, the Gompertz model fitted the BioCH_4_ system more than the first-order kinetic model. Similar findings were reported in previous studies^35,36^.

The steady-state CSTR was used to study the influence of HRT on the BioCH_4_ production. During the experiment, HRT was reduced from 10 to 1 d with the OLR escalating between 4.06 g_COD_/L.d^−1^ and 40.60 g_COD_/L.d^−1^. Principally, HRT and OLR play a critical role in the AD process, for their efficiency in substrate uptake and its tendency in determining the economics of the process^9^. The reaction failure during 10 d HRT was reasoned to the bad mixing in CSTR that caused the formation of a scum/dirt layer. Afterwards, a gradual improvement in the CSTR performance was observed with the reduction in HRTs; in terms of MPR, MY, VFAs consumption, COD and TSS removal (as shown in Fig. 2). According to Krishnan and co-authors, microorganisms overproduce BioCH_4_ when the system is subjected to a stepwise increase of OLR, then, they re-adjust their cell mechanisms depending on the newly applied process conditions, slowing down their metabolic activities to adapt to the new environment^37^.

Biochemical methane production (BMP) test was carried out to validate the selection of the optimum HRT for the system. Methane potential can specifically be expressed per amount of COD (L_CH4_/kg_COD_), at STP conditions; standard pressure (1 atm) and temperature (0 °C)^38^. Four HRT values were subjected to BMP test; 8, 5, 3 and 2 days, with mean experimental MY of 103.14, 120.05, 171.14 and 256.77 L_CH4_/kg_COD_, which represent 29.50%, 34.30%, 48.90% and 73.36%, respectively, of the theoretical MY (350 L_CH4_/kg_COD_)^39^. In this study, the optimum HRT that gave the closest MY to the theoretical MY was 2 d HRT. Additionally, lower HRT (higher OLR) might be beneficial for reducing reactor size^40^. In a previous study, an optimum HRT of 5 d recorded 85.6% of theoretical MY from POME at mesophilic conditions^41^. Also, AD of POME achieved 76–89% of theoretical methane potential at high VFA loading (1.8–4.7 g_VFA_/L), while 97% of theoretical methane potential was achieved for low VFA loading (0.9 g_VFA_/L)^42^.

Another important objective in this study was the removal of contaminants from the POME hydrogenic effluent. At the optimum HRT of 2 d, COD and TSS were deducted by 85.10% and 64.25%, respectively. Remarkably, the COD removal of the BioCH_4_ CSTR stage was much higher than that obtained in the BioH_2_ stage, signifying that most of the liquid effluent produced from the first-stage contained mostly soluble organic acids, which is more degradable and easier to convert to CH_4_ and CO_2_^26^. Two-stage AD have been proven to be more efficient than single stage AD. For instance, Mamimin and co-authors reported that TSS removal at single stage and two-stage methane production were 73% and 90% at HRT of 15 d and 17 d, respectively. This is due to the difficulty of degrading the complex content of raw POME by the microbial archaea in the single-stage^43^. In another study, more methane content (over 80%) was achieved from the evolved gases of the two-stage process than the methane content in the single-stage (50–75%) of cassava wastewater^26^.

Throughout the experiment, the dominant VFA was acetic acid followed by butyric acid, and negligible amount of propionic acid. This is due to the fact that all VFAs are first converted to acetic acid before being degraded to generate BioCH_4_, noting that the conversion rates of VFAs to BioCH_4_ varied based on the concentrations of acetic acid, ethanol, butyric acid, and propionic acid^42^. VFA is the most dominant intermediate during the anaerobic digestion process, however, the accumulation of VFA due to high OLR can lead to the end of the overall fermentation process^44^. The main focus of this study was to maintain the stability of CSTR performance with gradual increase in OLR, which was successful at HRTs of 8, 5, 3 and 2 days, as shown in Fig. 2(a,b). At HRT of 1 d (equivalent to OLR of 40.60 g_COD_/L.d^−1^), a drastic drop in the BioCH_4_ production occurred due to the overload of VFA and the microbial washout. According to Shi *et al*., VFA accumulation is the result of the imbalance between sequential steps of the AD process when methanogens cannot utilize hydrogen and VFA as quickly as they are produced by acidogens and acetogens. VFA accumulation can lead to rapid pH decrease and eventually process deterioration^45^. Therefore, it could be concluded that the best performance of the thermophilic BioCH_4_ in the CSTR that occurred at HRT of 2 d.

The acidogens and methanogens have different growth rates, nutritional requirements, and sensitivity to environmental changes. Thus, two-stage configuration of anaerobic digestion processes can optimize the environment for both kinds of microorganisms and improve the overall biogas production. Moreover, the acidogenic reactor can act as an effective buffer against sudden pH drops caused by accumulation of VFA. Therefore, two-stage anaerobic digestion systems are anticipated to have higher process reliability, resilience, stability, and higher substrate conversion^46^. The ratio of VFAs to total alkalinity (VFAs:TA) is an important parameter that can ease the monitoring of any biogas digester. It seems reasonable to postulate that the best VFAs:TA ratio is within the range of 0.23–0.40: higher than 0.40 led to reactor upset, and lower than 0.20 indicated that there was not enough substrate for the microbial archaea (i.e. microbial starvation)^9^. In this study, the system was most stable at HRT of 3 and 2 d. One of the advantages of two-stage anaerobic fermentation in this study is that the acetic acid in the hydrogenic POME effluent was the dominant VFA, whereas the formation of propionic acid in the first stage was less than 200 mM^28^. Thus, the methanogenic archaea were mainly the methane producing archaea from acetic acid. Similar reports corroborated that the increase of MPR occurred with the reduction in HRT alongside the increase of OLR, such as thermophilic biomethane production from acidified POME at HRT of 3 d^9^. On the other hand, studies on mesophilic BioCH_4_ production of two-stage anaerobic digestion using POME reported similar phenomenon as this study, but with higher HRTs; i.e. 5 d^47^, and 15 d^15,16^. According to Hamzah and co-authors, BioCH_4_ production under thermophilic conditions recorded higher productivity, faster stabilisation and higher COD removal than the mesophilic BioCH_4_ counterpart process^10^.

Microbial washout is a common drawback in CSTR systems; therefore, microbial granulation helps in overcoming this problem by keeping high biomass concentration inside the CSTR, even when operated at low HRT. Salem and co-authors have listed several operational parameters that must be controlled in order for the granulation to take place, including pH, temperature, OLR, substrate characteristics, nutrient feeding, microbial composition, etc^48^. In this study, small-sized MPGs were formed (Fig. 3a)), that could be related to the less solid content of the substrate as compared to the bigger-sized HPGs formed in the BioH_2_ reactor^28^. Furthermore, the shear force of the stirring impeller in CSTR does not support the granulation as compared to UASB bioreactors, which explains the hollow surface of the MPG, shown in Fig. 3(b,c). Zhang and his co-authors succeeded in producing biomass granules in an anaerobic CSTR in a short time despite the high mixing speed, by applying acid incubation approach on the culture, which enhanced the granulation process^49^. The tested EPS of MPGs resulted in a protein-to-carbohydrate ratio of 1.045, meaning that protein forms the majority of MPG. The physicochemical properties of MPGs were affected by the composition of EPS. The size of the MPG was relatively large compared to previous studies^50,51^, due to the high protein-to-carbohydrate ratio that was attributed to increased hydrophobicity and cohesion between aggregates^52^. According to Shin *et al*., high carbohydrate content in EPS increases the bioreactor’s sludge settleability^53^. Thus, in this study, sludge settling was low due to the continuous mixing, which led to less carbohydrate content in the EPS.

The balance between methanogens and acidogens is the key for a successful BioH_2_ and BioCH_4_ production, together with the sufficient soluble component available in the substrate for the anaerobic digestion. Notably, several molecular techniques such as terminal restriction fragment length polymorphism (T-RFLP)^54^, ribosomal intergenic spacer analysis (RISA)^55^, quantitative real-time PCR (q-PCR)^56^, fluorescent *in-situ* hybridization (FISH)^57^, denaturing gradient gel electrophoresis (DGGE)^18,32^ and next generation sequencing (NGS)^58^ have been extensively studied to identify the archaeal community population in biogas production systems. Although, NGS method has been recommended recently for the comprehensive analysis of bacterial community composition in heterogeneous environments. DGGE has been routinely used for the microbial identification of mixed cultures in anaerobic digestion systems, particularly for POME treatment^15,16,18^. Additionally, DGGE method requires simple and cheap equipment as compared to the recent methods^59^. Based on DGGE analysis in Fig. 4, *Methanothermobacter* sp. was the dominant genus at the optimum BioCH_4_ production conditions in this study. Similar genus was found in a methane sample from two-stage thermophilic *Chlorella* sp. biomass fermentation^60^, and POME samples with high loads of butyric acid and acetic acid that resulted in high BioCH_4_ production^42^. In fact, species belonging to *Methanothermobacter* sp. genus have been reported to be capable of autotrophic growth and were added to AD system to improve the syngas biomethanation process by enhancing the biomass conversion efficiency and the CH_4_ content of the evolved gases^61^. Similarly, *Methanbrevibacter* sp. was the dominant archaea in a two-stage thermophilic pilot-scale system for biohythane production from POME. This archaea is a thermophilic *Methanobacteriaceae* that is usually found in BioCH_4_ producing systems^18,32^. Moreover, *Methanobrevibacter* was reported to be acid-tolerant, which could grow in an acid rich environment, herein the hydrogenic POME effluent, at the pH of 5.0–7.5^44^. *Methanobacterium*, hydrogenotrophic methanogen, relies on H_2_/CO_2_ and formate as carbon sources and belongs to the class *Methanobacteria*^62^. *Methanobacterium* was reported to be one of the abundant archaea found in a microbial electrolysis cell-anaerobic digestion system, which contributed to a 2.8 times higher MY as compared to the conventional single stage anaerobic digestion^63^.

The calculated net energy gain *E*_*n*_ of BioCH_4_ producing CSTR system was *E*_*n*_ = 7.6 kJ/g_COD_ based on Eq. (4), the consumption energy included the energy needed for heating and mixing, while the generated energy was theoretically calculated from MY. The initial temperature was considered to be 45 °C, assuming a 10 °C drop in the effluent temperature due to the settling and feeding time, noting that the effluent from the DF-stage was at temperature of 55 °C. As a result, the total net energy gain for the two-stage anaerobic system was 11.2 kJ/g_COD_, including the DF- stage that yielded in 2.45 mol-H_2_/mol-sugar (equivalent to *E*_*n*_ = 3.6 kJ/g_COD_)^28^. Overall, the net energy gain of the two-stage system was a positive value. According to Perrera and co-authors, the *E*_*n*_ value declined with the increase of fermentation temperature and was less than 0 when the fermentation temperature exceeded 30 °C^64^. Hence, this study shows an improvement over reports of one stage biogas production above ambient-temperatures. The utilization of POME as substrate for thermophilic anaerobic digestion has an added advantage, since the mill discharge temperature is between 80 and 90 °C, and does not require high heating energy as compared to other substrates that need to be heated from ambient temperature before the commencement of the digestion process. More energy yield, 11.6 kJ/g_COD_, was obtained by O-Thong and co-authors^16^ for two reasons; first, the second stage AD was conducted at mesophilic temperature (35 °C) meaning that less energy was required for system’s heating. Second, a recirculation of BioCH_4_ effluent to the BioH_2_ fermenter was implied to keep control of the first stage’s temperature and pH. The energy yield of a similar study with a different substrate achieved 9.2 kJ/g_COD_ when treating *Agave tequilana* in mesophilic two-stage fermentation system^65^.

Table 2 shows the efficiency and productivity of the two-stage anaerobic system of this study compared to previous studies conducted earlier on various lignocellulosic wastes. The lowest HRT was successfully attained in this study together with high yields and production rates for both BioH_2_ and BioCH_4_, indicating that the thermophilic anaerobic system was stable and no microbial washout occurred in both stages. For two-stage anaerobic systems treating POME, the HRT for BioCH_4_ production stage applied in previous reports were; 10 d in UASB^18^, 15 d in UASB^15^, 5 d in CSTR^47^ and 8 d in microbial electrolysis cell (MEC)^37^. While the shortest HRT for producing BioCH_4_ from POME was recorded by this study, 2 d HRT, this could be related to the self-granulation of the sludge that enhanced the system’s stability, the pre-treatment of POME using diluted nitric acid which improved the first-stage process^28^, and the thermophilic temperature which is proven to increase the MPR when compared to mesophilic conditions^10^. Lay and co-authors achieved a lower HRT of 1 d, however, MY (58 L_CH4_/kg_COD_) was much lower than this study, since the substrate is a low-strength wastewater with low COD content^66^. Similarly, using olive pulp waste as the substrate for the two-stage AD resulted in low MY^67^. Thermophilic conditions were applied in two-stage systems to treat two different substrates; skim latex at HRT of 8 d^68^ and de-sugared molasses at HRT of 3 d^69^, although good MPR and MY were achieved, POME recorded almost more than double the MY compared to these two substrates. These findings can further support the viability of POME in minimizing the dependency on non-renewable energy sources at the mill, which adds more value to the palm oil industry besides CPO and CPKO.

**Table 2:** Comparison on the efficiency of two-stage BioH_2_ and BioCH_4_ production performance from various feedstock at different conditions.

Source	First stage					Second stage					Ref.
	Reactor	T (°C)	HRT (h)	HY* (mol_H2_/mol_sugar_)	HPR (L_H2_/L.d^−1^)	Reactor	T (°C)	HRT (d)	MY L_CH4_/kg_COD_	MPR (L_CH4_/L.d^−1^)	
POME	ASBR (35 L)	55	48	0.49	2.05	UASB (175 L)	55	10	265.12	1.90	^18^
POME	ASBR (0.2 L)	55	48	1.80	1.8	UASB (3 L)	35	15	315.00	2.6	^15^
POME	UASB (5 L)	55	48	1.80	2.15	CSTR (20 L)	37	5	310.00	3.2	^47^
POME	CSTR (0.8 L)	55	48	1.76	1.73	MEC (2.2 L)	37	8	290.00	2.7	^37^
Skim latex	UASB (1.35 L)	55	36	0.39	—	UASB (2.8 L)	55	8	130.70	—	^68^
Organic waste	CSTR (0.2 L)	55	72	—	0.22	CSTR (0.76 L)	55	12.6	—	1.47	^70^
De-sugared molasses	UASB (1 L)	55	16	0.53	5.6	UASB (4.5 L)	55	3	120	3.4	^69^
Olive pulp	CSTR (0.5)	55	28.7	1.24	0.15	CSTR (3 L)	35	10	98	1.13	^67^
Beverage wastewater	UASB (1 L)	35	8	0.01	—	UASB (1 L)	55	1	58	—	^66^
Pre-treated POME**	UASB (20 L)***	**55**	**6**	**2.45**	**2.93**	**CSTR (30 L)**	**55**	**2**	**256.77**	**4.3**	**This** study

*The total sugar consumed as glucose equivalent.

**Pre-treated POME with dilute nitric acid^71^.

***Biohydrogen production in the first-stage anaerobic fermentation^28^.

## Methods

### Seed inoculum and substrate

Thermophilic anaerobic seed sludge was obtained from palm oil mill anaerobic pond located at Sime Darby’s KKS Tennamaram, Bestari Jaya, Malaysia. The sludge was initially acclimatised in a 1-L Schott Duran bottle containing hydrogenic POME effluent for three months. When biogas produced did not exceed 10% variation, which was defined as stable production, the effluent samples were collected and used as seed for BioCH_4_ production in a 30-L CSTR.

Hydrogenic POME effluent was obtained from the BioH_2_ fermentation reactor fed with pre-treated POME (dilute nitric acid pre-treatment)^71^. The hydrogenic POME effluent was of brown-yellowish colour with a strong sour smell at the pH between 4.8 and 5.2, temperature of 55 °C, total COD of 63.27 ± 5.2 g/L, VFA of 3.25 ± 0.75 g CaCO_3_/L, TSS of 17.5 ± 2.0 g/L and VSS of 8.85 ± 1.5 g/L. Upon usage, the substrate’s pH was adjusted to pH 7.0 with 1 M KOH; the reaction was carried out at the same temperature (55 °C) as the first-stage reactor.

### Experimental Setup and the monitoring of CSTR

**Figure 5:**
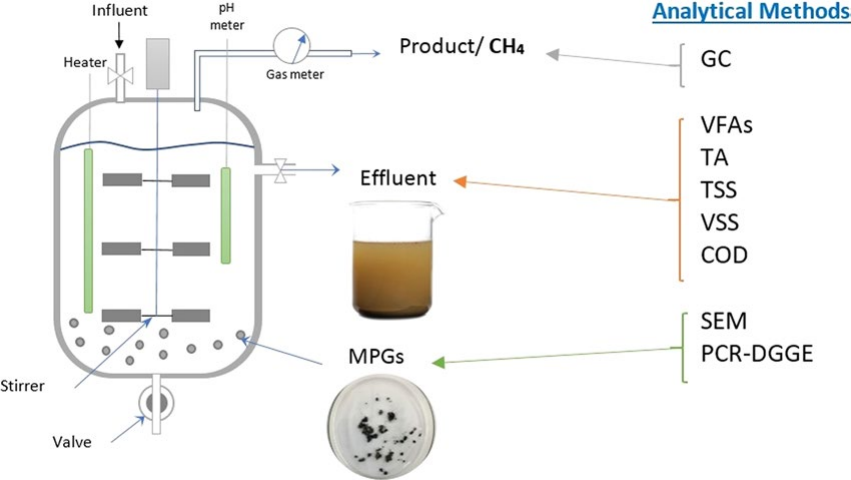
Schematic diagram of 30-L BioCH_4_ CSTR and the analytical methods applied in the study.

A schematic illustration of a 30-L CSTR with a working volume of 25 L is shown in Fig. 5. The bioreactor was initially filled with 5 L of an acclimatised seed inoculum. The working volume of the bioreactor was maintained throughout the whole experiment, in accordance with the design of the CSTR and the stable input and output streams. A multiple-impeller stirrer run by a stirring motor (200 watt, 3000 r/min) (Oriental Motor, Japan) provided a homogeneous mixing in the bioreactor. Once the biogas started to evolve during the start-up period, the reactor was subsequently fed with hydrogenic POME effluent at 2.5 L/d (equivalent to OLR of 4.06 g_COD_/L.d^−1^). The CSTR was operated by varying the HRTs from 10 d to 1 d, with the temperature maintained at 55 °C via a heating coil dipped inside the reactor. A wet gas flowmeter (Ritter, Germany) was installed at the gas outlet to record the volume of biogas produced. The system was assumed to be at steady-state when the biogas production was at 5% variation.

CSTR was routinely monitored for the pH, biogas production and composition, volatile fatty acids (VFAs) distributions, chemical oxygen demand (COD) and total suspended solids (TSS). The stabilized conditions with pH over 7.3 and VFAs:Alkalinity ratio less than 0.4 was confined to the methanogenic reactor. Table 3 outlines the CSTR’s feeding conditions at different HRTs. In order to avoid the loading-shock in the system, OLR was slowly increased stepwise to avoid microbial washouts. The pH of the feed (hydrogenic POME effluent) was set at 7.0–7.3 using 1 M KOH. N_2_ gas was purged at the bottom of the reactor to maintain anaerobic condition.

**Table 3:** Experimental setup for 30-L CSTR.

HRT (d)	Feeding mode (L/d)	OLR (g_COD_/L.d^−1^)
10	2.50	4.06
8	3.13	5.075
5	5.00	8.12
3	8.33	13.50
2	12.50	20.30
1	(12.5 × 2)* = 25	40.60

*Feeding twice per day.

### Analytical methods

The liquid effluent samples were analysed for their total VFAs, alkalinity and TSS based on standard methods^72^, and the COD was measured using_COD_ HACH kit (DR 2800, HACH). The composition of the biogas produced was monitored regularly, determined by gas chromatography (GC, Model SRI 8600 C, USA) equipped with a thermal conductivity detector (TCD) and a helium ionization detector (HID). The carrier gas used for the GC was helium (99.99% purity) at a flow rate of 25 mL/min. The initial oven temperature of the GC was set at 40 °C and 2.7 psi. The composition of VFAs was analysed by a UV detector HPLC (Agilent 1100, California, USA) in ROA column with a mobile phase of 0.005 N H_2_SO_4_ running at a flow rate of 0.6 mL/min.

The developed methane producing granules (MPGs) in the CSTR were viewed under field emission scanning electron microscopy (FESEM) (Supra 550VP, Germany). MPGs were fixed overnight in glutaraldehyde (2% (w/w)) at 4 °C. The fixed granules were washed three times with 0.1 M phosphate buffer saline (PBS) solution. Ethanol was used for dehydration of MPGs at concentrations of 30% to 90% for 10 min each time, and ended with three times washing in 100% (w/w) ethanol. The dehydrated MPGs were placed into a Critical Point Dryer (Leica Microsystems EM CPD 300, Germany) for 1.5 h. Dried MPGs were sputter-coated with platinum for FESEM viewing. Extracellular polymeric substances (EPS) was extracted using the formaldehyde-NaOH method from a previous study^73^, and the protein content of EPS was determined by Bradford method^74^.

### Kinetic study

The kinetics of the batch anaerobic digestion in the CSTR were examined with Eqs. (1) and (2). The Modified Gompertz Model (Eq. 1) was used to fit the cumulative BioCH_4_ production curve^75^ using the SigmaPlot Software v11.0 (Systat Software Inc., USA).

1 $$M(t)={M}_{0}\cdot exp\{\,-\,\exp [\frac{{R}_{m}e}{{M}_{0}}(\lambda -t)+1]\}$$

The First-order kinetics model^36^ was also used to fit the experimental data for BioCH_4_ gas in batch digester (Eq. 2),

2 $$M(t)={M}_{0}[1-\exp (\,-\,{K}_{G}^{{\prime} }\times t)]$$

where *M(t)*= Cumulative BioCH_4_ production from the experimental results (mL. at 1 atm, 25 °C), *M*_0_ = BioCH_4_ production potential (mL), *R*_*m*_= Maximum BioCH_4_ production rate (mL/h), *λ* = lag phase time (h), ${K}_{G}^{{\prime} }$ = the kinetic constant for BioCH_4_ production (per hour) that includes the biomass concentration, as shown in Eq. (3):

3 $${K}_{G}^{{\prime} }={K}_{G}\times X$$

*K*_*G*_ = the specific methane production kinetic constant (mL_CH4_/g VSS.h), and *X* = the biomass concentration (g/L VSS).

### Microbial analysis

#### Sample collection, DNA extraction, and PCR

In order to perform a polymerase chain reaction-denaturing gradient gel electrophoresis (PCR-DGGE) study on BioCH_4_ sludge, a sample was collected from the CSTR at HRT of 2 d and stored in a freezer at −20 °C. FavorPrep Soil DNA Isolation Mini Kit (Favorgen, USA) was used for DNA extraction. The DNA-extracted sample was amplified using the following PCR primers; 21F (TTCCGGTTGATCCYGCCGGA) and 958R (YCCGGCGTTGAMTCCAATT). Then, the sample was subjected to a nested-PCR run with 340F (CCTACGGGGYGCASCAG) with GC-Clamp (CGCCCGCCGCGCCCCGCGCCCGGCCCGCCGCCCCCGCCCCCC) and 519R (TTACCGCGGCKGCTG)^76^. The PCR component (PCR Master Mix, Promega, USA) was performed according to manufacturer instructions. The amplifications of samples were conducted using Eppendorf Mastercycler (Eppendorf AG, Germany).

#### DGGE and DNA sequencing

The nested PCR product was separated using VS20WAVE -DGGE (Cleaver Scientific, UK) on a vertical gel of 10% (w/v) acrylamide with a denaturant concentration of 30% (top) to 60% (bottom) of the gel. After loading the samples, electrophoresis was carried out in 1x TAE buffer at 145 V for 4.5 hours at 60 °C. The polyacrylamide gel was stained with SYBRR Green Nucleic Acid Gel Stain for 20 min then visualised and sliced out on Gel Imaging (FireReader V10, Uvitec, UK). The sliced gel bands were kept in 1x TE buffer overnight at 4 °C, then they were PCR-amplified with primer without the GC clamp. The samples were sent to 1^st^ Base Sdn. Bhd. for 16S rRNA sequencing. The obtained sequences were analysed by Basic Local Alignment Search Tool (BLAST) on National Centre for Biotechnology Information (NCBI) website. Finally, the BLAST analysed sequences were used to construct phylogenetic tree using the UPGMA method^77^ in MEGA software version 7.0^78^.

### Net energy gain (*E*_*n*_)

The theoretical net energy gain (*E*_*n*_) (kJ/g_COD_) was calculated as shown in Eq. (4)^64^, that is defined as the total energy produced equivalent to the volume of gas generated, minus the heat energy required to raise the reactor contents from ambient temperature to the fermentation temperature and the mixing energy consumed by the continuous stirring (*E*_*m*_),

4 $${E}_{n}=\frac{G{\rho }_{g}(LHV)-V{\rho }_{w}{C}_{\rho }({T}_{f}-{T}_{a})-{E}_{m}}{VC}$$

where, *G* is the biogas volume (L); *ρ*_g_ is CH_4_ gas density (7.16 × 10^−4^ kg/L); *LHV* is the lower heating value of CH_4_ (50,050 kJ/kg); *V* is the working volume of the bioreactor (L); *ρ*_*w*_ is the water density (1 kg/L); *C*_*ρ*_ is the water specific heat (4.2 kJ/kg K); *T*_*f*_ is the temperature at which the reaction took place (K); *T*_*a*_ the ambient temperature (K); *C* is the feed’s COD concentration (g_COD_/L); and *E*_*m*_ is the energy consumed by the stirrer’s motor (0.4 kWh).
